# Evolving Clinicopathological Characteristics of Women with Locally Advanced Cervical Cancer Treated with Definitive Chemoradiation Before and After Implementation of Organized Cervical Cancer Screening in Serbia

**DOI:** 10.3390/cancers18172891

**Published:** 2026-09-07

**Authors:** Jelena Stanić, Marija Popović-Vuković, Predrag Nikić, Ivana Šović, Luka Jovanović, Predrag Petrašinović, Marko Jovanović, Tatjana Arsenijević, Aleksandar Tomašević

**Affiliations:** 1 Faculty of Medicine, University of Belgrade, 11000 Belgrade, Serbia; maja1083@gmail.com (M.P.-V.); nicksha@gmail.com (P.N.); ppetrasinovic@yahoo.com (P.P.); tatjanaarsenijevic96@gmail.com (T.A.); alektom@gmail.com (A.T.); 2 Department of Radiation Oncology, Institute for Oncology and Radiology of Serbia, 11000 Belgrade, Serbia; 3 Clinic of Urology, University Clinical Center of Serbia, 11000 Belgrade, Serbia; 4 Department of Radiological Diagnostics, Institute for Oncology and Radiology of Serbia, 11000 Belgrade, Serbia; ivana90sovic@gmail.com

**Keywords:** locally advanced cervical cancer, definitive chemoradiation, organized cervical cancer screening, clinicopathological characteristics, FIGO 2018, Serbia

## Abstract

**Background:** Organized cervical cancer screening aims to reduce the burden of advanced disease through earlier detection. However, a substantial proportion of women continue to present with locally advanced cervical cancer (LACC) requiring definitive chemoradiation. This study evaluated temporal changes in the demographic and clinicopathological characteristics of women with LACC treated with definitive chemoradiation before and after implementation of the organized cervical cancer screening program in Serbia. **Methods:** This retrospective single-center cohort study included 200 consecutive women with histologically confirmed LACC treated with definitive chemoradiation at the Institute of Oncology and Radiology of Serbia. Two cohorts were analyzed: a pre-screening cohort (2010–2011, *n* = 100) and a post-screening cohort (2022–2023, *n* = 100). Demographic and clinicopathological characteristics, including age, histopathology, FIGO stage, lymph node involvement, and geographic distribution, were compared. For study purposes, all patients were retrospectively restaged according to the FIGO 2018 classification using the best available clinical, radiological, and pathological data. **Results:** Women in the post-screening cohort were significantly older at diagnosis than those in the pre-screening cohort (mean age 54.9 vs. 49.7 years, *p* = 0.0046), with a substantially higher proportion of patients older than 64 years (28% vs. 2%, *p* < 0.0001). Although the overall distribution of FIGO stages II–IV did not differ significantly, a marked redistribution of FIGO 2018 substages was observed (*p* < 0.0001), characterized by an increased proportion of stage IIIC disease and significantly more frequent lymph node involvement (63% vs. 41%, *p* = 0.0018). Geographic distribution remained stable, with most patients referred from the Belgrade administrative district. **Conclusions:** This study demonstrated clinically relevant temporal changes in the demographic and clinicopathological characteristics of women requiring definitive chemoradiation for LACC in Serbia. More than a decade after implementation of organized cervical cancer screening, tertiary referral centers continue to manage a substantial burden of patients with locally advanced disease requiring complex, resource-intensive treatment. These findings should not be interpreted as a direct evaluation of the national screening program but may provide valuable insights for healthcare planning in Serbia and other countries with similarly resource-constrained healthcare systems. Strengthening participation in organized screening, ensuring timely diagnostic evaluation, and improving HPV vaccination uptake remain essential to reduce the burden of advanced cervical cancer.

## Simple Summary

Cervical cancer can often be prevented or detected early through regular screening, but many women are still diagnosed when the disease requires intensive treatment with radiotherapy and chemotherapy. Serbia introduced an organized cervical cancer screening program in 2012. In this study, we compared 100 women treated for locally advanced cervical cancer before the introduction of organized screening (2010–2011) with 100 women treated more than a decade later (2022–2023). Women in the later period were older, more often had cancer involving the lymph nodes, and more frequently had disease classified as stage IIIC. These differences may partly reflect improvements in imaging and changes in cervical cancer staging over time. Our findings show that locally advanced cervical cancer continues to place an important burden on specialized cancer centers and highlight the need to improve participation in screening, timely diagnosis, and human papillomavirus vaccination.

## 1. Introduction

Despite being one of the most preventable malignancies, cervical cancer remains a major cause of cancer-related morbidity and mortality among women worldwide. According to GLOBOCAN 2022 estimates, approximately 662,000 women were diagnosed with cervical cancer and nearly 349,000 died from the disease, making it the fourth most frequently diagnosed cancer and the fourth leading cause of cancer-related death among women globally [1]. Although substantial progress has been achieved through human papillomavirus (HPV) vaccination and organized cervical cancer screening, cervical cancer continues to disproportionately affect low- and middle-income countries, where access to preventive healthcare and participation in screening programs remain suboptimal [1,2,3].

Locally advanced cervical cancer (LACC) remains one of the greatest therapeutic challenges in gynecologic oncology. Despite advances in cervical cancer prevention, a substantial proportion of women present with locally advanced disease requiring definitive chemoradiation, particularly in low- and middle-income countries and regions with incomplete screening coverage or delayed diagnosis [3,4,5,6]. Definitive chemoradiation, consisting of external beam radiotherapy (EBRT) with concurrent platinum-based chemotherapy followed by image-guided adaptive brachytherapy (IGABT), represents the standard treatment for patients with LACC who are not candidates for primary surgery and is recommended by all contemporary international guidelines [5,6,7,8,9]. The implementation of modern radiotherapy techniques, including intensity-modulated radiotherapy (IMRT), image-guided adaptive brachytherapy, and advanced imaging for treatment planning, has substantially improved local tumor control while reducing treatment-related toxicity [5,6,7,8,9,10,11]. Nevertheless, treatment outcomes remain strongly influenced by treatment quality, tumor stage, lymph node involvement, overall treatment time, and timely access to specialized multidisciplinary care [3,4,5,6,7,8,9,10,11].

Accurate assessment of lymph node status has become an integral component of contemporary staging and treatment planning in LACC. The introduction of the FIGO 2018 staging system recognized the prognostic importance of pelvic and para-aortic lymph node metastases by incorporating nodal involvement into stage IIIC disease, thereby influencing treatment selection, radiotherapy planning, and expected clinical outcomes [4,5,12].

Women requiring definitive chemoradiation represent a distinct subgroup within the cervical cancer population, characterized by the highest treatment complexity and the greatest demand for radiotherapy resources. These patients require prolonged multimodality treatment, advanced radiotherapy technologies, repeated imaging, and coordinated multidisciplinary management. Consequently, temporal changes in the demographic and clinicopathological characteristics of women referred for definitive chemoradiation may provide valuable insights into evolving disease presentation, optimization of treatment strategies, and future healthcare planning [5,6,7,8,9,10,11].

Persistent infection with high-risk HPV genotypes represents the necessary etiological factor for the development of virtually all cervical cancers [2,3]. Therefore, effective prevention relies on two complementary strategies: primary prevention through HPV vaccination and secondary prevention through organized cervical cancer screening [2]. Countries that have successfully implemented population-based screening programs have demonstrated substantial reductions in cervical cancer incidence, mortality, and the proportion of advanced-stage disease through early detection and treatment of precancerous lesions [2,13,14,15,16]. However, the effectiveness of these programs depends largely on screening coverage, adherence to recommended screening intervals, equitable access to healthcare services, and timely diagnostic evaluation of abnormal findings [2,14,15,16].

In Serbia, an organized population-based cervical cancer screening program was introduced nationwide in December 2012 and targets women aged 25–64 years [17]. The program is based on conventional cervical cytology (Pap smear), which is recommended every three years for women with normal findings [17,18]. Women with abnormal cytological findings are referred for further diagnostic evaluation according to national screening protocols. In recent years, cervical cancer prevention activities in Serbia have been gradually modernized through the introduction of HPV testing alongside cervical cytology in selected preventive initiatives, representing an important step toward broader incorporation of HPV-based testing into cervical cancer screening practice [18,19].

Primary prevention has also been strengthened through implementation of HPV vaccination. Since June 2022, the nonavalent HPV vaccine has been available free of charge in Serbia for children and adolescents aged 9–19 years through the national immunization program [20,21]. However, HPV vaccination uptake remains relatively low, with considerable regional variation across the country [20]. Despite these important preventive initiatives, participation in organized cervical cancer screening remains suboptimal, and cervical cancer continues to represent a considerable public health burden in Serbia [18,22].

Most previous studies have evaluated the impact of organized cervical cancer screening on cervical cancer incidence, mortality, and stage distribution at the population level [13,14,15,16]. In contrast, few studies have focused specifically on temporal changes in the demographic and clinicopathological characteristics of women with LACC requiring definitive chemoradiation in routine clinical practice. Interpretation of such changes is further complicated by advances in diagnostic imaging, pathological reporting, FIGO staging, and radiotherapy techniques over the past decade [5,6,7,8,9,10,12].

Therefore, the aim of this study was to evaluate temporal changes in the demographic and clinicopathological characteristics of women with locally advanced cervical cancer treated with definitive chemoradiation before and after implementation of the organized cervical cancer screening program in Serbia, with particular attention to age distribution, histopathological characteristics, FIGO stage, lymph node involvement, and geographic distribution. The study was not designed to directly evaluate the effectiveness of the national cervical cancer screening program.

## 2. Methods

### 2.1. Study Design

This retrospective single-center cohort study was conducted at the Institute of Oncology and Radiology of Serbia, the largest tertiary referral center for radiation oncology and gynecologic malignancies in Serbia. The study compared two temporal cohorts of women with locally advanced cervical cancer treated with definitive chemoradiation: 100 consecutive patients treated between January 2010 and December 2011, before implementation of the organized national cervical cancer screening program, and 100 consecutive patients treated between January 2022 and December 2023, approximately ten years after its introduction.

### 2.2. Patient Selection

Eligible patients were identified through institutional treatment records and electronic medical databases. One hundred consecutive patients per time period who met the predefined eligibility criteria were included in the analysis.

The study population comprised women with histologically confirmed locally advanced cervical cancer who were selected for definitive concurrent chemoradiation following multidisciplinary tumor board evaluation. Histopathological diagnosis, including histological subtype and tumor grade, was established before treatment using cervical biopsy specimens obtained according to routine institutional diagnostic practice, typically by targeted cervical punch biopsy, with endocervical sampling when clinically indicated. This approach is consistent with standard diagnostic recommendations for cervical cancer [5]. As all patients included in the study were selected for definitive chemoradiation rather than primary surgery, surgical resection specimens were not expected to be available for histopathological assessment. Nevertheless, limited biopsy material may not fully capture intratumoral heterogeneity, particularly with respect to tumor grading.

Patients treated primarily with surgery, those receiving palliative treatment, patients with recurrent cervical cancer, and patients with incomplete clinical or pathological data required for the planned analyses were excluded. The same inclusion and exclusion criteria were applied to both cohorts.

### 2.3. Data Collection

Clinical and pathological data were retrospectively extracted from institutional medical records. Data collection, verification, and statistical analysis were performed between April and July 2026 following approval by the Ethics Committee of the Institute of Oncology and Radiology of Serbia. Collected variables included age at diagnosis, place of residence, histopathological subtype, tumor grade, FIGO stage and substage, lymph node involvement, and localization of lymph node metastases. Geographic distribution was analyzed according to patients’ administrative district of residence.

Because the study compared patients treated more than a decade apart, diagnostic work-up reflected routine clinical practice during the respective study periods. Consequently, the availability and utilization of modern imaging modalities differed between the two cohorts. In particular, pelvic magnetic resonance imaging (MRI) was not routinely performed in most patients treated during 2010–2011, whereas contemporary imaging protocols, including routine MRI, were implemented in the later cohort. This reflects the evolution of routine diagnostic practice in Serbia during the study period and should be considered when interpreting temporal changes between the two cohorts. During the earlier study period, assessment of parametrial invasion relied predominantly on clinical gynecological examination, consistent with the FIGO staging recommendations in use at that time.

Furthermore, HPV genotyping was not routinely performed during the earlier study period and, despite its increasing use in recent years, is still not systematically incorporated into routine diagnostic practice in Serbia. Likewise, p16 immunohistochemistry was not consistently available across both cohorts. The absence of systematic HPV and p16 data represents a limitation of the present study, as it precluded reliable classification of tumors according to HPV-associated or HPV-independent etiology and assessment of potential temporal changes in HPV-related tumor biology.

### 2.4. Restaging According to FIGO 2018

To ensure uniform comparison between the two cohorts, for the purpose of this study, all patients were retrospectively restaged according to the FIGO 2018 classification using all available clinical, radiological, and pathological information documented in the medical records. The retrospective restaging was performed solely for study purposes and did not influence the treatment originally delivered. When MRI findings were unavailable in the earlier cohort, restaging was based on the best available clinical, radiological, and pathological documentation recorded at the time of diagnosis.

### 2.5. Statistical Analysis

Continuous variables were summarized using means and standard deviations or medians with ranges, as appropriate. Categorical variables were presented as absolute numbers and percentages.

Comparisons between the two cohorts were performed using the Wilcoxon rank-sum test for continuous variables and the chi-square test or Fisher’s exact test for categorical variables, as appropriate. All statistical tests were two-sided, and *p* values < 0.05 were considered statistically significant. Statistical analyses were performed using IBM SPSS Statistics software (Version 29.0; IBM Corp., Armonk, NY, USA).

Because this was a retrospective exploratory cohort study, including all consecutive eligible patients treated during the predefined study periods, no *a priori* sample size calculation was performed. The sample size was determined by the total number of eligible patients available in the institutional database. Given the exploratory nature of the study, no adjustment for multiple testing was performed.

### 2.6. Ethical Considerations

The study was approved by the Ethics Committee of the Institute of Oncology and Radiology of Serbia (Approval No. 0101/2026/1108). Owing to the retrospective design and the use of anonymized clinical data, the requirement for informed consent was waived by the Ethics Committee. The study was conducted in accordance with the ethical principles of the Declaration of Helsinki.

## 3. Results

A total of 200 women with locally advanced cervical cancer were included in the analysis, comprising 100 patients in the pre-screening cohort (2010–2011) and 100 patients in the post-screening cohort (2022–2023).

### 3.1. Demographic and Clinicopathological Characteristics

**Figure 1:**
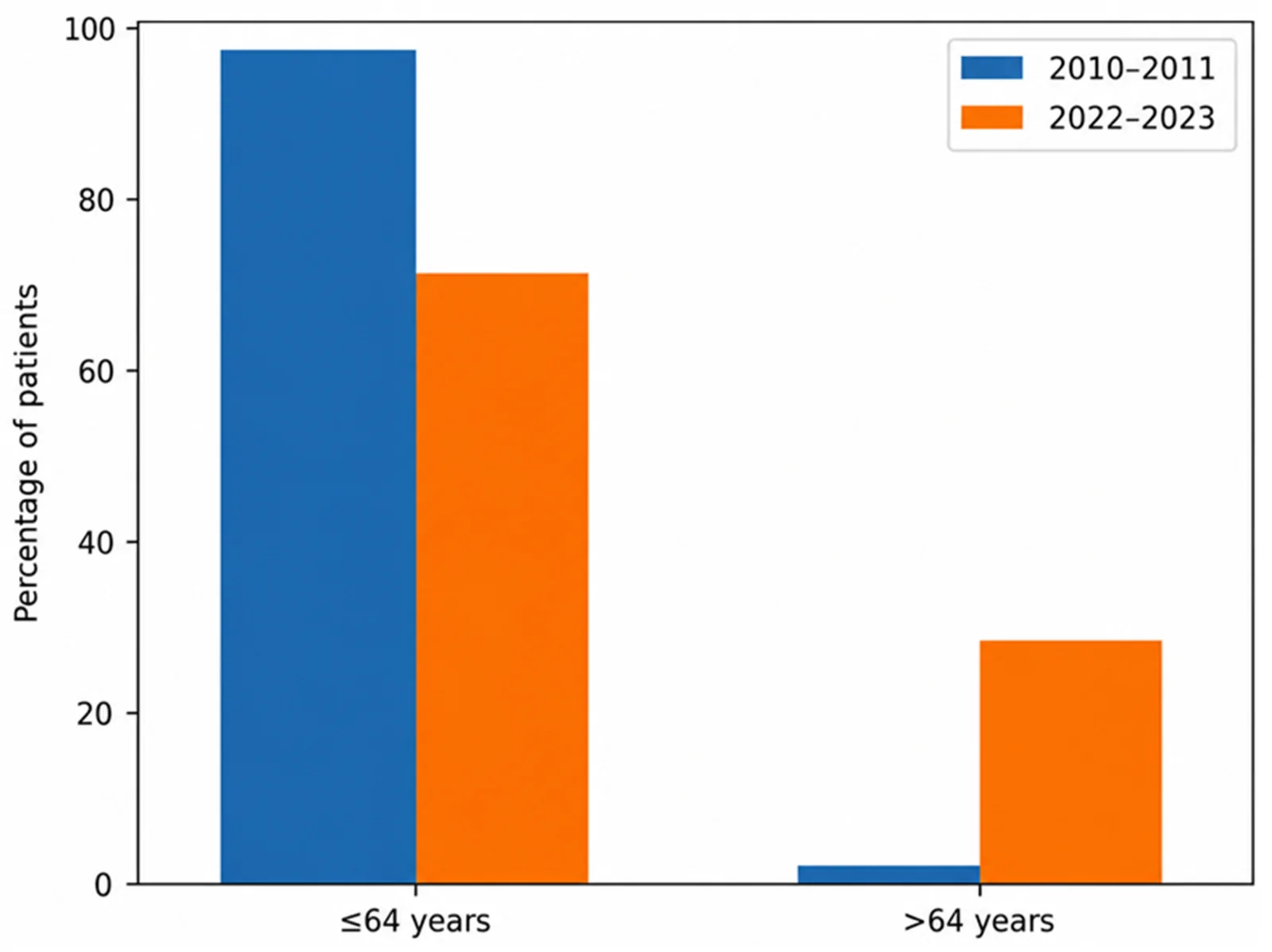
Age distribution according to screening eligibility in the pre-screening (2010–2011) and post-screening (2022–2023) cohorts.

Women in the later cohort were significantly older at diagnosis than those treated in 2010–2011 (mean age, 54.9 vs. 49.7 years; median age, 56 vs. 51 years; *p* = 0.0046). The proportion of women older than 64 years increased markedly from 2% to 28% (*p* < 0.0001) (Figure 1). Comorbidities were more frequent in the later cohort, although the difference did not reach statistical significance (54% vs. 42%; *p* = 0.089).

Squamous cell carcinoma was the predominant histological subtype in both cohorts. The distribution of histological cancer subtypes differed significantly between the study periods (*p* = 0.046), whereas tumor grade distribution remained similar (*p* = 0.253). Lymph node involvement was significantly more frequent in the post-screening cohort than in the pre-screening cohort (63% vs. 41%; *p* = 0.0018), whereas the distribution of lymph node localization among node-positive patients remained comparable between the two cohorts (*p* = 0.64) (Table 1).

### 3.2. Temporal Changes in FIGO Stage Distribution

**Figure 2:**
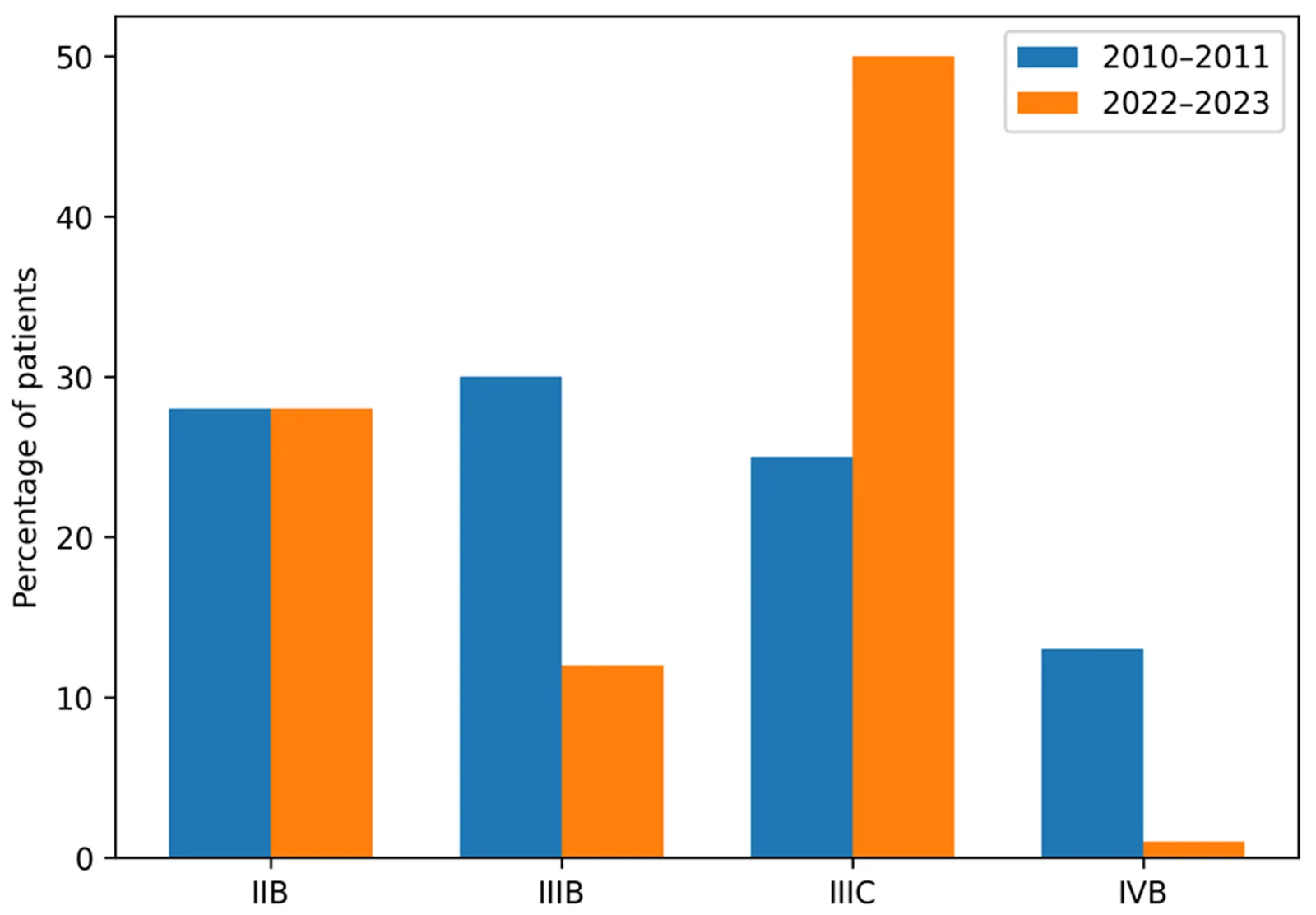
Temporal changes in the distribution of the predominant FIGO 2018 substages in the pre-screening (2010–2011) and post-screening (2022–2023) cohorts. Complete FIGO substage distribution is presented in Table 1.

Although no significant difference was observed in the overall distribution of FIGO stages II–IV between the two cohorts (*p* = 0.14), analysis according to the FIGO 2018 staging system revealed a significant redistribution across individual substages over time (*p* < 0.0001). The proportion of patients with FIGO stage IIB remained unchanged (28% in both cohorts). In contrast, the frequency of FIGO stage IIIC increased from 25% in the pre-screening cohort to 50% in the post-screening cohort. Conversely, the proportions of patients with FIGO stage IIIB and IVB decreased from 30% to 12% and from 13% to 1%, respectively. A modest increase was observed in the proportion of patients with FIGO stage IVA disease (3% vs. 7%), whereas FIGO stage IIA remained uncommon in both cohorts (Table 1, Figure 2).

Among patients with advanced disease (FIGO stages III–IV), the proportion of women older than 64 years was significantly higher in the post-screening cohort than in the pre-screening cohort (27.1% vs. 3.2%; *p* = 0.0001) (Table 1).

### 3.3. Geographic Distribution of Patients

The majority of patients in both cohorts resided in the Belgrade administrative district, accounting for 63.0% of the pre-screening cohort and 58.0% of the post-screening cohort. The Mačva administrative district represented the second largest referral region in both cohorts (14.0%), while the proportion of patients referred from the Podunavski administrative district increased from 5.0% to 13.0% over time. Patients from several other administrative districts of Serbia, as well as a small number from neighboring countries, were also represented. Overall, no statistically significant difference in geographic distribution was observed between the two study periods (Fisher’s exact test, *p* = 0.156) (Table 2).

## 4. Discussion

In this retrospective single-center cohort study, we evaluated temporal changes in the demographic and clinicopathological characteristics of women with locally advanced cervical cancer treated with definitive chemoradiation before and after implementation of the organized national cervical cancer screening program in Serbia. The principal findings of the study were a significant increase in the proportion of women older than 64 years, higher rates of lymph node involvement, and a marked shift toward FIGO stage IIIC disease in the later cohort. These findings should be interpreted in the context of substantial changes in cervical cancer diagnosis, staging, and treatment over the past decade, as well as the gradual implementation of cervical cancer prevention strategies in Serbia.

Although organized cervical cancer screening has been implemented in Serbia since 2012, women requiring definitive chemoradiation for locally advanced cervical cancer continued to represent an important clinical population at our institution during both study periods. The observed findings should therefore be interpreted as temporal changes within this specific clinical population rather than as changes in the overall incidence or stage distribution of cervical cancer in Serbia [17,18].

The geographic distribution of patients remained stable throughout the study period, with most women originating from the Belgrade administrative district and surrounding regions. This finding is expected because the Institute of Oncology and Radiology of Serbia is the largest national tertiary referral center for gynecologic oncology and radiation therapy, receiving patients from across the country. The absence of significant differences in geographic distribution between the two cohorts supports their comparability and suggests that the observed temporal changes are unlikely to be explained by major differences in referral patterns alone.

These referral patterns should also be considered in relation to the population distribution of Serbia. Belgrade, which contributed the largest proportion of patients in both cohorts, is also by far the most populous administrative area in the country [23]. Nevertheless, because the present study included only patients treated at our tertiary referral center, these figures represent institutional referral patterns and should not be interpreted as population-based incidence rates. Conversely, lower numbers of patients referred from individual areas cannot be assumed to indicate a lower burden of cervical cancer, as they may reflect differences in population size, referral pathways, geographic accessibility, and the availability of oncology services outside our institution.

At the same time, the persistence of a substantial burden of locally advanced cervical cancer among women treated at our institution is noteworthy, particularly considering that Belgrade has the greatest availability of specialized healthcare services, organized screening infrastructure, and diagnostic resources in Serbia. These findings suggest that important challenges in cervical cancer prevention and early diagnosis remain even in the most developed part of the country. Given the well-recognized regional differences in healthcare access, socioeconomic conditions, and participation in preventive programs, these challenges may be even more pronounced in less developed regions, although this could not be directly evaluated in the present study [20,22].

One of the most notable findings of the present study was the significant shift in FIGO substage distribution, particularly the increase in FIGO stage IIIC disease from 25% to 50% in the later cohort. Several factors may have contributed to this observation. First, advances in diagnostic imaging over the past decade have substantially improved assessment of both local tumor extent and regional lymph node involvement. During the earlier study period, pelvic magnetic resonance imaging (MRI) was not routinely performed in most patients, whereas it became an integral component of contemporary cervical cancer staging in the later cohort, allowing more accurate evaluation of parametrial invasion and local tumor extent. Likewise, the increasing availability of ^18^F-FDG PET/CT has improved detection of pelvic and para-aortic lymph node metastases, resulting in more accurate nodal staging and treatment planning [24,25]. Second, the introduction of the FIGO 2018 staging system fundamentally changed cervical cancer classification by incorporating pelvic and para-aortic lymph node metastases as stage IIIC1 and IIIC2 disease, respectively [5,12]. Importantly, FIGO 2018 allows lymph node involvement to be determined by imaging or pathological assessment. Accordingly, lymph node status in the present study was based on the diagnostic imaging available at initial evaluation. However, imaging-based nodal assessment cannot provide definitive histopathological confirmation of metastatic involvement and may be affected by false-positive findings, including reactive or inflammatory lymphadenopathy. Therefore, the higher proportion of lymph node-positive and FIGO stage IIIC patients in the later cohort may partly reflect improved nodal detection and imaging-related stage migration rather than a true increase in nodal metastatic disease. Overall, the observed temporal changes should be interpreted as the combined effect of evolving diagnostic practice, modifications in staging criteria, and possible changes in disease presentation over time.

The high prevalence of lymph node involvement observed in both cohorts is also consistent with previously published studies of locally advanced cervical cancer. Prospective and retrospective studies have demonstrated that pelvic lymph node metastases are present in a substantial proportion of patients with FIGO stage IIB–IIIB disease, while para-aortic lymph node involvement is observed in a clinically relevant subset of patients [26,27]. Since lymph node status represents one of the strongest prognostic factors in cervical cancer, accurate identification of nodal metastases has become increasingly important for treatment stratification, target volume delineation, and selection of appropriate radiation fields [12,27]. The higher proportion of lymph node-positive patients observed in the later cohort is therefore likely to reflect improvements in diagnostic staging rather than an isolated change in tumor biology.

Another important finding of the present study was the significant increase in the proportion of women older than 64 years in the later cohort. This observation is particularly noteworthy because the organized cervical cancer screening program in Serbia primarily targets women aged 25–64 years [17,18]. Consequently, women older than 64 years are no longer routinely invited to participate in the organized screening program after reaching the upper age limit, particularly if they have not previously participated regularly. Similar concerns have been reported in other European countries, emphasizing the importance of adequate screening participation before program exit and appropriate follow-up of women at increased risk [14,15]. The marked increase in women older than 64 years in the later cohort may therefore warrant further investigation of screening exit criteria and preventive strategies for older women, particularly those with a history of inadequate or irregular screening participation.

In addition, demographic changes, increasing life expectancy, and improvements in general health status may have contributed to a greater number of older women being considered suitable candidates for definitive chemoradiation. Although the present study was not designed to determine the causes of this age shift, the findings highlight the need for continued evaluation of cervical cancer prevention strategies and clinical management in older women.

The present findings should also be interpreted in the context of ongoing cervical cancer prevention efforts in Serbia. Organized population-based cervical cancer screening has been implemented nationwide since 2012, and according to the most recent national report, more than 62,000 women underwent organized screening during 2024, with over 3300 referred for additional diagnostic evaluation because of abnormal screening findings [18]. Furthermore, pilot implementation of HPV testing combined with cervical cytology has demonstrated the feasibility of gradually introducing HPV-based screening into routine clinical practice in Serbia [18]. Although these findings indicate continuous development and modernization of the national screening program, participation remains below the desired level, and previous studies have identified limited public awareness and insufficient participation in preventive programs as important barriers to successful cervical cancer prevention [19,22]. In the present study, although the distribution of histological cancer subtypes differed between the two cohorts, HPV-associated and HPV-independent tumors could not be reliably distinguished because HPV genotyping and p16 immunohistochemistry were not systematically available across both study periods. Therefore, the potential contribution of HPV-related tumor biology to the observed temporal differences cannot be determined from the available data. Despite this limitation, the Serbian experience may provide useful insights for other healthcare settings in which systematic HPV genotyping and p16 assessment are not routinely available. In such settings, interpretation of temporal changes in cervical cancer characteristics should consider not only the implementation of screening programs but also concurrent changes in diagnostic, pathological, and staging practices.

Primary prevention has also been strengthened through introduction of free HPV vaccination for children and adolescents; however, vaccine uptake remains relatively low despite nationwide availability [20]. Importantly, HPV vaccination was introduced only shortly before inclusion of patients in the later cohort and therefore could not reasonably be expected to influence the clinicopathological characteristics observed in the present study. Nevertheless, experiences from countries with high vaccination coverage have demonstrated substantial reductions in cervical cancer precursors and cervical cancer incidence, emphasizing the importance of improving vaccine acceptance and maintaining high participation in organized screening as complementary strategies for long-term cervical cancer control [28,29,30].

Oncological and radiotherapy services in Serbia are provided through a limited network of specialized institutions, including the Institute for Oncology and Radiology of Serbia in Belgrade, the Oncology Institute of Vojvodina in Sremska Kamenica, and regional oncology and radiotherapy centers in Kragujevac, Niš, and Kladovo [31]. Referral pathways are influenced by the territorial organization of the national healthcare system and the availability of specialized services. Importantly, these centers differ in their diagnostic and therapeutic capacities, available equipment, and human resources, which may also influence referral patterns. The relatively limited number and unequal distribution of radiotherapy centers should therefore be considered when interpreting the geographic distribution of patients in the present study.

Taken together, our findings indicate that a substantial burden of locally advanced cervical cancer requiring definitive chemoradiation persists more than a decade after the introduction of organized screening in Serbia. Although the present study was not designed to evaluate screening effectiveness, these findings underscore the importance of high screening participation, timely diagnostic work-up, broader implementation of HPV-based testing, adequate access to specialized oncological care, and effective primary prevention strategies. The marked increase in the proportion of women older than 64 years requiring definitive chemoradiation also raises the question of whether current screening exit criteria and preventive strategies for older women should be further evaluated, particularly among those with inadequate or irregular prior screening.

## 5. Strengths

The present study has several important strengths. To our knowledge, this is the first study from Serbia to evaluate temporal changes in the demographic and clinicopathological characteristics of women with locally advanced cervical cancer treated with definitive chemoradiation before and after implementation of the organized national cervical cancer screening program. An important methodological strength is the inclusion of a homogeneous study population comprising exclusively women referred for and treated with definitive chemoradiation following multidisciplinary tumor board evaluation. Restricting the analysis to patients with a uniform treatment indication minimized heterogeneity related to treatment selection and avoided direct comparison of clinically different populations managed with primary surgery, definitive chemoradiation, or palliative treatment.

All consecutive eligible patients treated during two predefined study periods were included, and identical inclusion and exclusion criteria were applied to both cohorts. This approach reduced the potential for investigator-driven selection of cases and strengthened the internal validity of the temporal comparison. Furthermore, all patients were treated at the Institute of Oncology and Radiology of Serbia, the largest tertiary referral center for gynecologic oncology and radiation therapy in the country, using institutional treatment protocols and multidisciplinary decision-making. The inclusion of patients referred from several administrative districts also provides insight into real-world patterns among women requiring definitive chemoradiation in a major national oncology center.

## 6. Limitations

Several limitations should be acknowledged. First, the retrospective single-center design precludes causal inference and limits generalizability of the findings. Second, individual information regarding participation in organized or opportunistic cervical cancer screening, HPV vaccination status, socioeconomic characteristics, and health-seeking behavior was unavailable, preventing assessment of their direct influence on disease presentation. Third, important advances in diagnostic imaging occurred during the study period. Although all patients were retrospectively restaged according to the FIGO 2018 classification using the best available documentation, differences in imaging availability, particularly the routine use of pelvic MRI in the later cohort, may have contributed to stage migration. In addition, lymph node status was based on diagnostic imaging without systematic histopathological confirmation; therefore, imaging-related false-positive or false-negative nodal findings cannot be excluded.

Another limitation was the lack of systematic HPV genotyping and p16 immunohistochemistry data across both cohorts, which precluded reliable classification of tumors according to HPV-associated or HPV-independent etiology.

In addition, histopathological characterization was based on pretreatment cervical biopsy specimens rather than surgical resection specimens. Although this reflects standard diagnostic practice in patients undergoing definitive chemoradiation, limited biopsy material may not fully capture intratumoral heterogeneity, particularly with respect to tumor grading, and may therefore reduce the precision of histopathological comparisons between the two cohorts.

Finally, the study intentionally included only women treated with definitive chemoradiation. This design provided a homogeneous study population but limits extrapolation of the findings to women with early-stage cervical cancer treated primarily with surgery. Evaluation of survival outcomes was beyond the scope of the present study, which was designed to investigate temporal changes in patient and tumor characteristics rather than treatment outcomes.

## 7. Conclusions

This retrospective single-center study demonstrated clinically relevant temporal changes in the demographic and clinicopathological characteristics of women with locally advanced cervical cancer treated with definitive chemoradiation at a national tertiary referral center in Serbia. Compared with the pre-screening cohort, women treated during the later period were older, more frequently presented with lymph node involvement, and showed a marked shift toward FIGO stage IIIC disease. These findings most likely reflect the combined influence of evolving diagnostic practice, implementation of the FIGO 2018 staging system, and temporal changes in disease presentation over the past decade.

Importantly, the present findings should not be interpreted as a direct evaluation of the effectiveness of the organized cervical cancer screening program. Rather, they describe temporal changes among women requiring definitive chemoradiation for locally advanced cervical cancer. More than a decade after the introduction of organized cervical cancer screening in Serbia, a substantial proportion of women referred to a tertiary oncology center still require definitive chemoradiation, reflecting the persistent burden of locally advanced disease on the healthcare system. Given that definitive chemoradiation is a complex, multidisciplinary, and resource-intensive treatment requiring advanced radiotherapy technology, concurrent chemotherapy, image-guided brachytherapy, and specialized expertise, these findings emphasize the continuing need to strengthen cervical cancer prevention and early detection.

From a public health perspective, our findings highlight that greater emphasis should be placed on primary healthcare and preventive strategies, including improving participation in organized screening, ensuring timely diagnostic evaluation of abnormal findings, and increasing HPV vaccination uptake. Early detection and prevention are not only associated with better patient outcomes but may also reduce the need for complex and costly treatment delivered in tertiary referral centers.

Although this study reflects the experience of a national tertiary referral center in Serbia, the findings may also be relevant to other countries with limited healthcare resources and a persistently high burden of locally advanced cervical cancer. Our experience suggests that, even after implementation of organized screening, continued investment in prevention, early diagnosis, and primary healthcare remains essential to reduce the demand for resource-intensive treatment and optimize allocation of healthcare resources.

## Figures and Tables

**Table 1:** Demographic and clinicopathological characteristics of patients in the pre-screening and post-screening cohorts.

Characteristic	2010–2011 (*n* = 100)	2022–2023 (*n* = 100)	Total (*n* = 200)	*p*-Value
Demographic characteristics				
Age at diagnosis (years)	Mean ± SD: 49.7 ± 10.0	Mean ± SD: 54.9 ± 13.0	Mean ± SD: 52.3 ± 11.9	*p* = 0.0046
	Median (range): 51 (26–73)	Median (range): 56 (29–80)	Median (range): 53 (26–80)	
Age group according to screening eligibility (years)	≤64: 98 (98%)	≤64: 72 (72%)	≤64: 170 (85%)	χ^2^ = 22.2,
	>64: 2 (2%)	>64: 28 (28%)	>64: 30 (15%)	*p* < 0.0001
Comorbidities	Yes: 42 (42%)	Yes: 54 (54%)	Yes: 96 (48%)	χ^2^ = 2.88,
	No: 58 (58%)	No: 46 (46%)	No: 104 (52%)	*p* = 0.089
Clinicopathological characteristics				
Histopathology	Squamous: 83 (83%)	Squamous: 92 (92%)	Squamous: 175 (87.5%)	*p* = 0.046
	Adenocarcinoma: 10 (10%)	Adenocarcinoma: 8 (8%)	Adenocarcinoma: 18 (9%)	
	Adenosquamous: 3 (3%)	Adenosquamous: 0 (0%)	Adenosquamous: 3 (1.5%)	
	Other: 4 (4%)	Other: 0 (0%)	Other: 4 (2%)	
Tumor grade	G1: 12 (12%)	G1: 19 (19%)	G1: 31 (15.5%)	χ^2^ = 2.75,
	G2: 65 (65%)	G2: 53 (53%)	G2: 118 (59%)	*p* = 0.253
	G3: 14 (14%)	G3: 15 (15%)	G3: 29 (14.5%)	
	Unknown: 9 (9%)	Unknown: 13 (13%)	Unknown: 22 (11%)	
Lymph node involvement	Yes: 41 (41%)	Yes: 63 (63%)	Yes: 104 (52%)	χ^2^ = 9.70,
	No: 59 (59%)	No: 37 (37%)	No: 96 (48%)	*p* = 0.0018
Lymph node localization *	Pelvic: 25 (61.0%)	Pelvic: 38 (60.3%)	Pelvic: 63 (60.6%)	*p* = 0.64
	Para-aortic: 2 (4.9%)	Para-aortic: 2 (3.2%)	Para-aortic: 4 (3.8%)	
	Inguinal: 0 (0%)	Inguinal: 3 (4.8%)	Inguinal: 3 (2.9%)	
	Combined: 14 (34.1%)	Combined: 20 (31.7%)	Combined: 34 (32.7%)	
FIGO substage	IIA: 0 (0%)	IIA: 2 (2%)	IIA: 2 (1%)	*p* < 0.0001
	IIB: 28 (28%)	IIB: 28 (28%)	IIB: 56 (28%)	
	IIIA: 1 (1%)	IIIA: 0 (0%)	IIIA: 1 (0.5%)	
	IIIB: 30 (30%)	IIIB: 12 (12%)	IIIB: 42 (21%)	
	IIIC: 25 (25%)	IIIC: 50 (50%)	IIIC: 75 (37.5%)	
	IVA: 3 (3%)	IVA: 7 (7%)	IVA: 10 (5%)	
	IVB: 13 (13%)	IVB: 1 (1%)	IVB: 14 (7%)	
Additional analysis				
Age group among patients with advanced disease (FIGO III–IV)	≥64: 2 (3.2%)	≥64: 19 (27.1%)	≥64: 21 (15.8%)	*p* = 0.0001
	<64: 61 (96.8%)	<64: 51 (72.9%)	<64: 112 (84.2%)	

Legend: Data are presented as mean ± standard deviation (SD), median (range), or *n* (%), as appropriate. Comparisons between the pre-screening (2010–2011) and post-screening (2022–2023) cohorts were performed using the Wilcoxon rank-sum test, chi-square test, or Fisher’s exact test, as appropriate. * Percentages for lymph node localization were calculated only among patients with lymph node metastases (2010–2011: *n* = 41; 2022–2023: *n* = 63; total: *n* = 104). The analysis of age distribution according to FIGO stage included only patients with advanced disease (FIGO stages III–IV).

**Table 2:** Geographic distribution of patients by administrative district in the pre-screening and post-screening cohorts.

Administrative District	2010–2011 (*n* = 100)	2022–2023 (*n* = 100)	Total (*n* = 200)
Belgrade	63 (63%)	58 (58%)	121 (60.5%)
Mačva	14 (14%)	14 (14%)	28 (14%)
Podunavski	5 (5%)	13 (13%)	18 (9%)
Južno-Banatski	6 (6%)	8 (8%)	14 (7%)
Kolubarski	2 (2%)	3 (3%)	5 (2.5%)
Montenegro	4 (4%)	0 (0%)	4 (2%)
Šumadijski	0 (0%)	1 (1%)	1 (0.5%)
Kosovsko-Mitrovački	0 (0%)	1 (1%)	1 (0.5%)
Sremski	1 (1%)	1 (1%)	2 (1%)
Moravički	1 (1%)	0 (0%)	1 (0.5%)
Pčinjski	1 (1%)	0 (0%)	1 (0.5%)
Bosnia and Herzegovina	0 (0%)	1 (1%)	1 (0.5%)
Zlatiborski	2 (2%)	0 (0%)	2 (1%)
Rasinski	1 (1%)	0 (0%)	1 (0.5%)

Legend: Data are presented as *n* (%). Overall geographic distribution did not differ significantly between the two cohorts (Fisher’s exact test, *p* = 0.156).

## Data Availability

The datasets generated and/or analyzed during the current study are not publicly available because they contain confidential patient information but are available from the corresponding author upon reasonable request and with permission of the Institute of Oncology and Radiology of Serbia.

## Abbreviations

The following abbreviations are used in this manuscript:

CRT	Chemoradiation
EBRT	External Beam Radiotherapy
FIGO	International Federation of Gynecology and Obstetrics
HPV	Human Papillomavirus
IGABT	Image-Guided Adaptive Brachytherapy
IMRT	Intensity-Modulated Radiotherapy
IORS	Institute of Oncology and Radiology of Serbia
LACC	Locally Advanced Cervical Cancer
MRI	Magnetic Resonance Imaging
PET/CT	Positron Emission Tomography/Computed Tomography
